# Regulation of Heart Rate in *Drosophila* via Fragile X Mental Retardation Protein

**DOI:** 10.1371/journal.pone.0142836

**Published:** 2015-11-16

**Authors:** Stefanie Mares Novak, Archi Joardar, Carol C. Gregorio, Daniela C. Zarnescu

**Affiliations:** 1 Department of Cellular and Molecular Medicine and Sarver Molecular Cardiovascular Research Program, The University of Arizona, Tucson, Arizona, 85724, United States of America; 2 Department of Molecular and Cellular Biology The University of Arizona, Tucson, Arizona, 85721, United States of America; CNRS UMR7275, FRANCE

## Abstract

RNA binding proteins play a pivotal role in post-transcriptional gene expression regulation, however little is understood about their role in cardiac function. The Fragile X (FraX) family of RNA binding proteins is most commonly studied in the context of neurological disorders, as mutations in Fragile X Mental Retardation 1 (FMR1) are the leading cause of inherited mental retardation. More recently, alterations in the levels of Fragile X Related 1 protein, FXR1, the predominant FraX member expressed in vertebrate striated muscle, have been linked to structural and functional defects in mice and zebrafish models. FraX proteins are established regulators of translation and are known to regulate specific targets in different tissues. To decipher the direct role of FraX proteins in the heart *in vivo*, we turned to *Drosophila*, which harbors a sole, functionally conserved and ubiquitously expressed FraX protein, dFmr1. Using classical loss of function alleles as well as muscle specific RNAi knockdown, we show that *Drosophila* FMRP, dFmr1, is required for proper heart rate during development. Functional analyses in the context of cardiac-specific *dFmr1* knockdown by RNAi demonstrate that dFmr1 is required cell autonomously in cardiac cells for regulating heart rate. Interestingly, these functional defects are not accompanied by any obvious structural abnormalities, suggesting that dFmr1 may regulate a different repertoire of targets in *Drosophila* than in vertebrates. Taken together, our findings support the hypothesis that dFmr1 protein is essential for proper cardiac function and establish the fly as a new model for studying the role(s) of FraX proteins in the heart.

## Introduction

RNA regulation provides a critical mechanism for controlling gene expression in the heart during normal development and disease. Yet post-transcriptional mechanisms of gene expression remain understudied in the heart. This area of investigation is particularly critical since it was reported that >70% of transcripts are subcellularly localized in *Drosophila* [1]. Additionally, in eukaryotes ~60% of proteins are estimated to be regulated post-transcriptionally [2,3]. In this study, we examine the role of the RNA binding protein, Fragile X Mental Retardation Protein 1 in the *Drosophila* heart. A majority of studies on the Fragile X (FraX) family of RNA binding proteins has focused on its role in inherited mental retardation, in particular Fragile X syndrome and autism [4,5]. However, it has been shown that loss of *Fxr1*, the predominant striated muscle family member in vertebrates, leads to perinatal lethality in mice most likely due to cardiac defects, while its reduction in zebrafish leads to disruption of cellular architecture, impaired cardiac function, and cardiomyopathies [6–8]. Despite the connection between FraX and cardiac function, the requirement for these proteins specifically in the heart has yet to be identified.

To better understand the role of FraX proteins in the heart, we turned to the genetic capabilities of *Drosophila*. In *Drosophila*, there is only one FraX protein (dFmr1) that is ubiquitously expressed [9]. It is homologous to FraX vertebrate proteins and harbors a high degree of homology within its RNA binding domains: two KH domains and a C-terminal RGG box [9]. Over the years, *Drosophila* has emerged as an excellent model system for studying the basic molecular and genetic mechanisms of cardiac development and function (for reviews see [10–13]). The *Drosophila* heart, also known as the dorsal vessel, is a muscular pump composed of an inner layer of contractile muscle cells (cardial cells) flanked by non-contractile pericardial cells (for review see [10]). With the *Drosophila* heart functioning as a pump for hemolymph, which carries necessary immune cells and nutrients to maintain homeostasis, it makes for a simple yet physiologically relevant model to study cardiac function. Tissue specific knockdown tools allow the study of mutations that may otherwise cause severe or fatal cardiac dysfunction and lethality in other model systems (for review see [14]).

Here, we report that dFmr1 is required for proper control of heart rate in the absence of obvious structural defects. We show that loss of dFmr1 function results in a significant decrease in heart rate that is rescued by a wild type copy of *dFmr1* expressed in its genomic context (*i*.*e*., genomic rescue) [9]. Importantly, our data show that dFmr1 is required cell autonomously in cardiac muscle as evidenced by a significant decrease in heart rate caused by cardiac-specific RNAi knockdown of *dFmr1* but not a neuronal- or glial-specific RNAi knockdown. Additionally, using a larval turning assay to analyze locomotor function, the cardiac-specific *dFmr1* RNAi knockdown results in slower rollover (*i*.*e*., increased larval turning times), consistent with impaired locomotion that accompanies cardiac dysfunction. Our findings establish a new, genetically tractable model of cardiac physiology, based on dFmr1 that could be exploited in the future to decipher novel signaling pathways governing heart function.

## Experimental Procedures

### Drosophila genetics

Fly stocks were maintained at 25°C on standard cornmeal agar medium. Transheterozygous larvae were produced by crossing *w* ^*1118*^ *; FRT82B dFmr1* ^*3*^ */TM6B GFP* (*i*.*e*., *dFmr1* ^*3*^ *)* with *w* ^*1118*^ *; FRT82B dFmr1* ^*50M*^ */TM6B GFP* (*i*.*e*., *dFmr1* ^*50M*^) provided by K. Broadie, Vanderbilt University. Genomic rescue of dFmr1 was performed by crossing *dFmr1* ^*50M*^ with *w* ^*1118*^ *; P[dFmr1]; dFmr1* ^*3*^ */TM6C* (stock provided by T. Jongens, University of Pennsylvania). For tissue-specific knockdown of dFMR1, *w* ^*1118*^ *; P[GD1288]v8933*, (*dFmr1* ^*RNAi-1*^; from Vienna *Drosophila* RNAi center) or *y* ^*1*^ *sc* ^*^ *v* ^*1*^ *; P[y* ^*+t7*.*7*^ *v* ^*+t1*.*8*^ *= TRiP*.*HMS00248]attP2*, (*dFmr1* ^*RNAi-2*^; from Bloomington *Drosophila* stock center) were crossed with the muscle-specific Gal4 driver *w; P[GawB]how* ^*24B*^ (24B Gal4, Bloomington *Drosophila* stock center), the cardiac-specific Gal4 driver *P[tinC-Gal4*.*Δ4]* (TinC Gal4, stock provided by R. Bodmer, Sanford Burnham Medical Research Institute), the neuronal-specific Gal4 driver *P{w[+mW*.*hs] = GawB}elav[C155]* (Elav Gal4, Bloomington *Drosophila* stock center) or the glia-specific Gal4 driver *w;repo-Gal4/TM6C* (Repo Gal4, Bloomington *Drosophila* stock center).

### Western blot analysis

Lysates from 3–5 third instar larvae were prepared in a modified Laemmli buffer [15]. Larvae were snap frozen in liquid nitrogen and processed at 60°C in lysis buffer (4M Urea, 1M Thiourea, 1.5% SDS, 37.5mM DTT, 0.025M Tris-HCl, 25% glycerol and 0.015% bromophenol blue) with 1x Halt protease inhibitor cocktail (Life Technologies). The expression of dFMR1 was detected using mouse anti-dFMR1 antibodies (6A15 at 1:1500, ab10299 from Abcam). Tubulin was used as a loading control and was detected using a mouse anti-tubulin antibody (KMX-1 at 1:2000, mab3408 from Millipore). Secondary antibody was HRP-conjugated donkey anti-mouse IgG (1:10,000, 715-036-150 from Jackson ImmunoResearch). After incubation in SuperSignal West Pico chemiluminescent substrate (Thermo Scientific), blots exposed on autorad film (Bioexpress) were developed (Konica film processor) and scanned (Epson perfection 4870 Photo scanner).

### Analysis of pre-pupal heart rate

Video microscopy to analyze and measure heart rate was done as previously described [16]. In brief, 60s videos were collected using a Zeiss dissecting microscope equipped with a digital camera (640x480 pixels at 30 frames s^-1^) (FinePix S9000, Fujifilm). Manual counts of heart rate were collected using Apple Quicktime Player at half speed and using a cell counter to calculate beats per minute. Heart rate analysis was performed on *dFmr1* null larvae and controls (*w* ^*1118*^). To analyze the tissue-specific effects of dFmr1, two independent *dFmr1* RNAi lines were analyzed using the pan-muscle 24B and cardiac specific TinC Gal4 drivers. Controls include the Gal4 drivers (24B and TinC) crossed with *w* ^*1118*^. Statistical analysis was performed with Prism 6 statistical software (GraphPad Software, Inc.) using unpaired, two-tailed Student’s t-test.

### Immunofluorescence

Semi-intact larval heart preparations were prepared according to a previously published method with a few modifications [17]. Prior to removing organs, the semi-intact larva was relaxed 15 min with artificial hemolymph (AH) (108mM NaCl, 5mM KCl, 2mM CaCl_2_, 8mM MgCl_2_, 1mM NaH_2_PO_4_, 4mM NaHCO_3_, 10mM Sucrose, 5mM Trehalose, 5mM HEPES pH 7.3) containing 10mM EGTA then fixed in 2% paraformaldehyde in AH for 15 min (*e*.*g*., [18]). Primary antibody: 6A15 against dFmr1 (1:500) (Abcam). Secondary antibody: Alexa Fluor 488-conjugated goat anti-mouse IgG (1:1,000) (Jackson Immunoresearch). Filamentous actin was detected with Texas Red-conjugated phalloidin (1:200) (Life Technologies) prior to mounting with Aqua Poly/Mount (Polysciences Inc.). Low magnification images were captured using a Deltavision RT system (Applied Precision) with a 40x NA 1.35 objective, a charge-coupled device camera (CoolSNAP HQ; Photometrics), and deconvolved using SoftWoRx software (Applied Precision). Higher magnification images were captured using a Leica SP5 confocal microscope with a 63x NA 1.4 objective. All images were processed using Photoshop CS (Adobe). Analysis of filamentous actin was performed using an intensity plot profile in the ImageJ 2.0 software (National Institutes of Health) and analyzed using Prism 6 statistical software (GraphPad Software, Inc.).

## Larval turning assay

Assays were performed as previously described [19]. Wandering third instar larvae were placed on a grape juice plate at room temperature. After becoming acclimated, crawling larvae were gently turned onto their backs (ventral side up), and monitored until they turned back (dorsal side up) and continued their forward movement. The time it took each larva to complete this task was recorded. Thirty larvae were used per genotype. Statistical analysis was performed with Prism 6 statistical software (GraphPad Software, Inc.) using unpaired, two-tailed Student’s t-test.

### Climbing assays

Flies were raised at 25°C. Ten (1-day-old) adult males of each genotype were collected and tested for their climbing ability starting at 1 day old and every 7 days thereafter, until they reached 22 days after eclosion. Five cohorts (50 males) were tested for each genotype. To assess their climbing ability, the flies were transferred to an empty vial marked 5 cm from the bottom. After being allowed to acclimate to the new environment (∼30s), flies were gently tapped down to the bottom of the vial and then the time it took each fly to pass the 5cm mark was recorded. All flies that climbed the 5cm up the vial in 18s or less passed, whereas those that could not climb that high or took longer than 18s failed. Both the number of flies that passed and the number of flies surviving were recorded each time the test was performed. The climbing index for each genotype was calculated as the number of flies that passed the climbing test, normalized to the number of survivors on the day of the test. Survivability was calculated by dividing the number of flies alive on each day by the number alive on day 1. Statistical analysis was performed with Prism 6 statistical software (GraphPad Software, Inc.) using a two-way ANOVA with multiple comparisons.

## Results

### Heart rate is regulated by *dFmr1* in *Drosophila*

**Fig 1:**
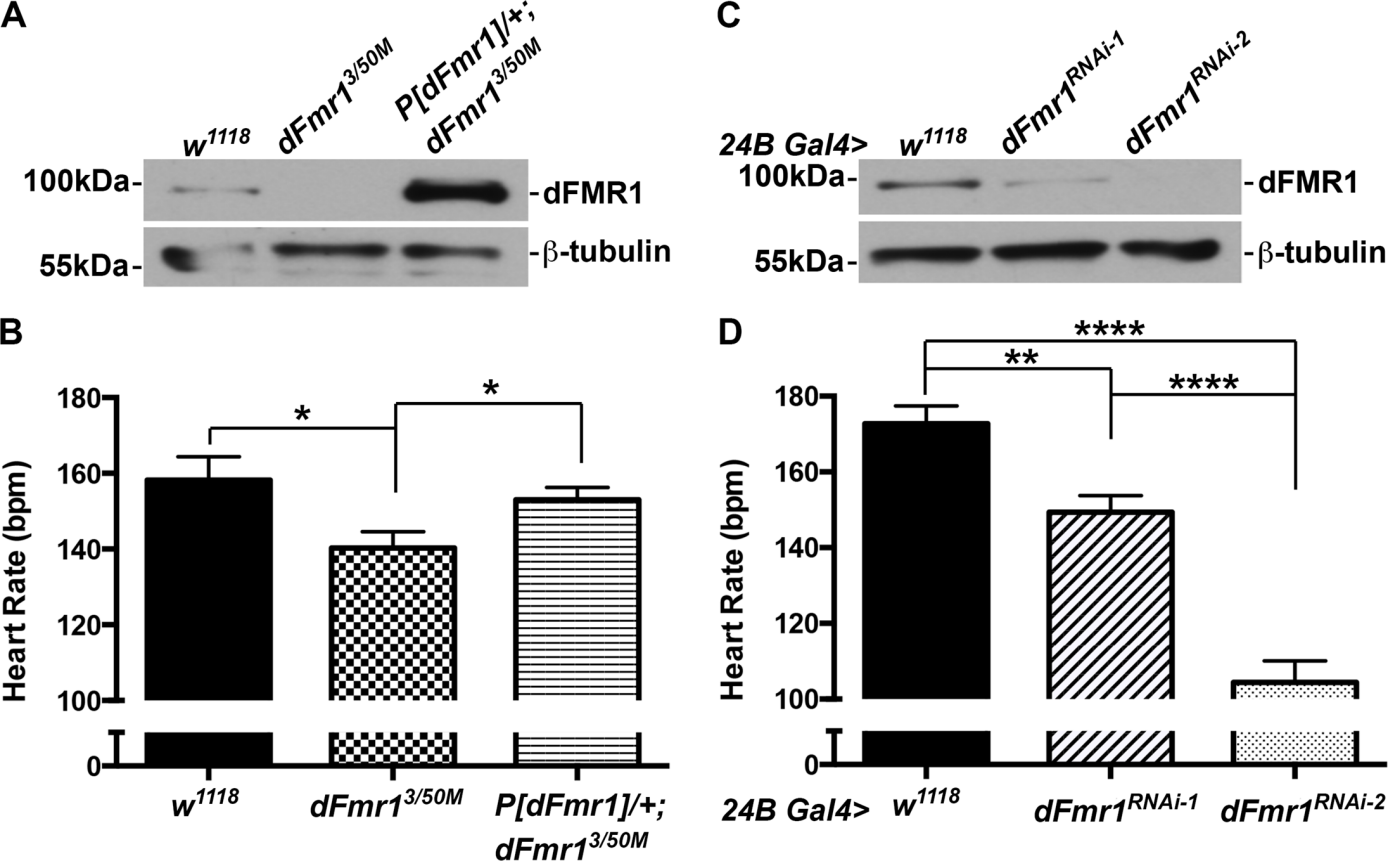
dFmr1 regulates heart rate. **(A)** Western blot analysis showing the complete knockout of dFmr1 (*dFmr1* ^*3/50M*^) compared to wild type (*w* ^*1118*^) and genomic rescue (*P[dFmr1]/+; dFmr1* ^*3/50M*^). **(B)** Loss of dFmr1 (*dFmr1* ^*3/50M*^, N = 16) results in a statistically significant decrease in heart rate when compared to wild type (*w* ^*1118*^, N = 10). This is rescued by expression of a wild type copy of *dFmr1* in its genomic context (*P[dFmr1]/+; dFmr1* ^*3/50M*^, N = 10). **(C)** Western blot showing that two independent RNAi lines (24B Gal4> *dFmr1* ^*RNAi-2*^, *dFmr1* ^*RNAi-2*^) result in almost complete striated muscle-specific knockdown of dFMR1 (i.e., ~85–100%) compared to control (24B Gal4> *w* ^*1118*^). **(D)** Pan-muscle (24B Gal4) RNAi knockdown of dFmr1 (24B Gal4> *dFmr1* ^*RNAi-1*^, N = 12 and *dFmr1* ^*RNAi-2*^, N = 6) leads to a significant decrease in heart rate compared to control (24B Gal4> *w* ^*1118*^, N = 17). Student’s T-test was used to calculate statistical significance: p values, * <0.05, ** <0.01, **** <0.0001.

To determine whether dFmr1 regulates cardiac function *in vivo*, we analyzed heart rate in *Drosophila* pupae. We first analyzed a transheterozygous mutant, *dFmr1* ^*3/50M*^, which has no detectable dFmr1 expression by western blot analysis (*dFmr1* null, **Fig 1A**). When compared to wild type controls (*w* ^*1118*^), the *dFmr1* null mutant results in a ~11% reduction in heart rate, which is statistically significant (158±6.2 vs. 140±4.3 beats per minute, P_value_ = 0.029, **Fig 1B**). To confirm that the decrease in heart rate was specifically caused by loss of dFmr1 and not by genetic background effects, we performed a genomic rescue experiment. When the genomic rescue construct *P[dFmr1]* was introduced in the *dFmr1* null background (*w* ^*1118*^ *;P[dFmr1]/+;dFmr1* ^*3/50M*^), dFmr1 expression (**Fig 1A**) and the heart rate were restored to wild type levels (153±3.2 beats per minute, **Fig 1B**).

We next analyzed the effect of *dFmr1* reduction on heart rate using RNAi knockdown. The Gal4-UAS bipartite system [20] was used to express two independent RNAi constructs (see Experimental Procedures) in all developing muscles with the pan-mesodermal 24B Gal4 driver [20] and found that both *dFmr1* RNAi lines cause a significant decrease in dFmr1 levels, with 24B Gal4> dFmr1^RNAi-1^ larvae exhibiting ~84% whereas 24B Gal4> dFmr1^RNAi-2^ larvae exhibit no detectable dFmr1 expression when compared to controls (24B Gal4> *w* ^*1118*^, **Fig 1C**). Notably, both RNAi lines result in a statistically significant decrease in heart rate; the RNAi-1 line resulted in a ~14% decrease, whereas the RNAi-2 line had a more dramatic decrease of ~40% when compared to controls (149±4.4 and 104±5.7 vs. 173±4.7 beats per minute, P_values_ = 0.0012 and 0.71E-06, **Fig 1D**). These results show that the extent to which FMRP expression is reduced correlates directly with the degree of contractile impairment. Overall, these data indicate that dFmr1 is required for proper control of heart function during development in *Drosophila*.

### *dFmr1* is autonomously required in cardiac cells to regulate heart rate

**Fig 2:**
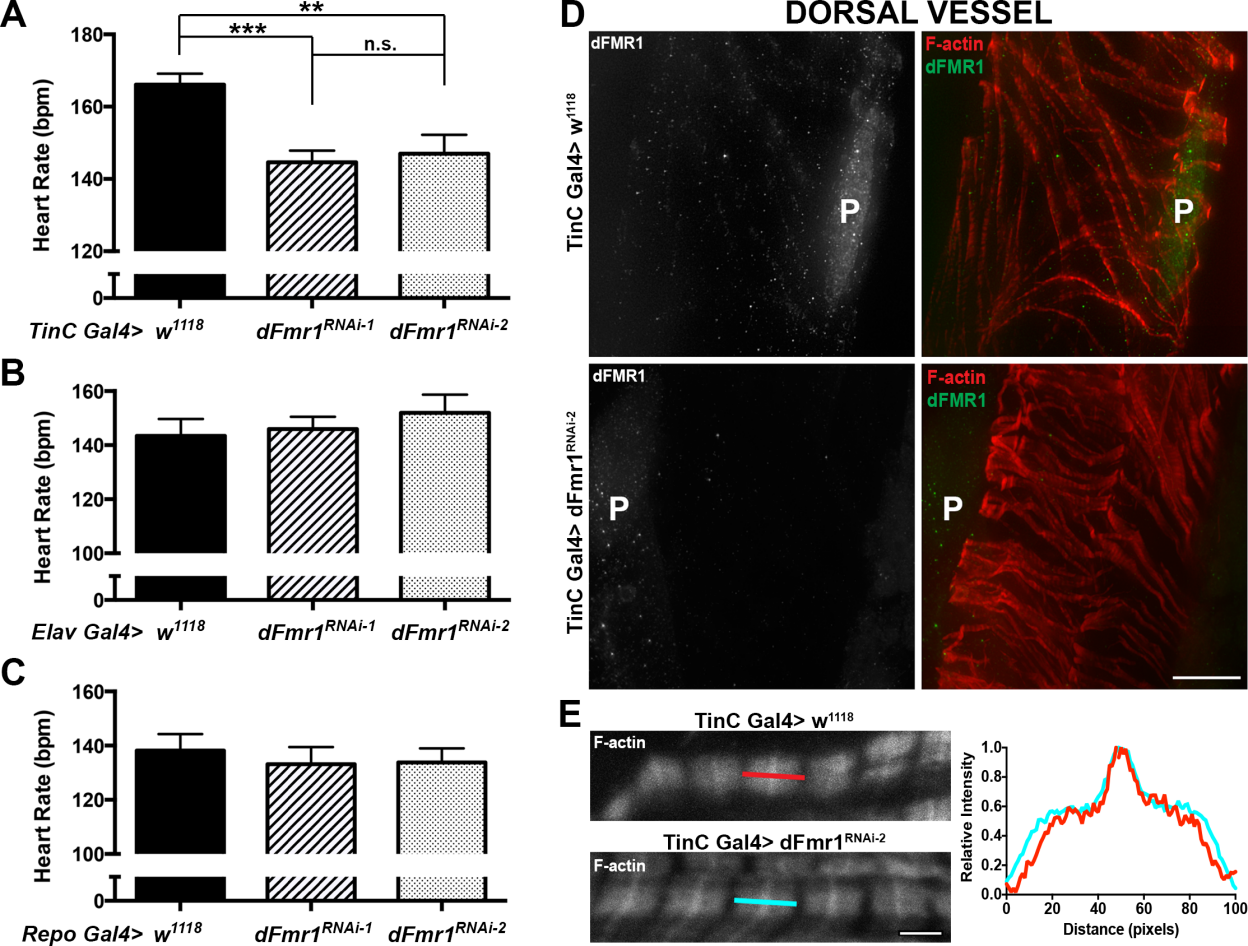
dFmr1 regulation of heart rate is cardiac-specific. **(A)** Heart muscle (TinC Gal4) RNAi knockdown of dFmr1 (TinC Gal4> *dFmr1* ^*RNAi-1*^, N = 10 and *dFmr1* ^*RNAi-2*^, N = 10) results in a significant decrease in heart rate compared to control (TinC Gal4> *w* ^*1118*^, N = 17). **(B)** Neuronal-specific (Elav Gal4) and **(C)** panglial (Repo Gal4) RNAi knockdown of dFmr1 (*dFmr1* ^*RNAi-1*^, N = 11 and *dFmr1* ^*RNAi-2*^, N = 11) do not result in a significant decrease in heart rate compared to control (*w* ^*1118*^, N = 11). Student’s T-test was used to calculate statistical significance: p values, n.s. = no significance, * <0.05, ** <0.01, *** <0.001. **(D)** Dorsal vessel of the RNAi knockdown of dFmr1 (TinC Gal4> *dFmr1* ^*RNAi-2*^, *dFmr1* ^*RNAi-2*^) stained with anti-dFmr1 (shown in green) results in a visible decrease in dFmr1 expression compared to control (TinC Gal4> *w* ^*1118*^, Control), as seen in the pericardial cells (P) and the cardiomyocytes. Scale bar = 20 μm. **(E)** No major changes in sarcomere structure as seen by staining for filamentous actin could be detected. Scale bar = 2 μm.

To determine whether the decrease in heart rate is cardiac muscle specific, we next analyzed the effects of *dFmr1* knockdown in the dorsal vessel. To this end, we expressed dFmr1^RNAi-1^ or dFmr1^RNAi-2^ in cardiomyocytes using the TinC Gal4 driver [21] and found that both resulted in a statistically significant decrease in heart rate. TinC Gal4> dFmr1^RNAi-1^ larvae exhibited ~13% decrease in heart rate (145±3.0 vs. 166±5.2 beats per minute, P_value_ = 0.74E-04, **Fig 2A**), which is similar to the results seen with the pan-mesodermal 24B Gal4 driver (**Fig 1D**). However, TinC Gal4> dFmr1^RNAi-2^ larvae exhibited only a ~12% decrease (147±3.3 beats per minute vs. 166±5.2 beats per minute, P_value_ = 0.0061, **Fig 2A**) when compared to controls (TinC Gal4> *w* ^*1118*^), which is less than the ~40% decrease seen with the 24B Gal4 driver (**Fig 1D**). This difference seen with dFmr1^RNAi-2^ when driven by the pan-mesodermal 24B Gal4 driver (~40% decrease) versus the cardiac TinC Gal4 driver (~12% decrease) could be due to a non-autonomous effect in the context of the pan-mesodermal driver 24B compared to the more restricted, cardiac specific TinC. Alternatively, dFmr1^RNAi-2^ may exhibit off-target effects in the context of 24B Gal4.

To confirm the change in heart rate was specific to reduction of dFMR1 in cardiomyocytes, we also examined the heart rate using additional Gal4 drivers. We expressed dFmr1^RNAi-1^ or dFmr1^RNAi-2^ in the central nervous system using the elav Gal4 driver [22] and in glial cells using the repo Gal4 driver [23]. Both the neuronal-specific and panglial RNAi knockdown of dFMR1 resulted in no significant change in heart rate compared with control (**Fig 2B and 2C**). Overall, our data show that dFmr1 knockdown by RNAi in cardiomyocytes leads to a significant decrease in heart rate, consistent with a cell autonomous role for dFmr1 protein in the dorsal vessel, in regulating cardiac function.

Immunofluorescence imaging of dFmr1 stained tissue confirms the knockdown of dFMR1 in the heart with a visible reduction of dFMR1 in the pericardial cells and the dorsal vessel (**Fig 2D**). Notably, fluorescently-conjugated phalloidin labeling of the filamentous actin in the heart shows no obvious defects in sarcomeric structure (**Fig 2E**). This suggests the alterations caused by dFmr1 loss on heart rate are likely not due to a major structural defect.

### Reduced cardiac function causes impaired locomotor behavior

**Fig 3:**
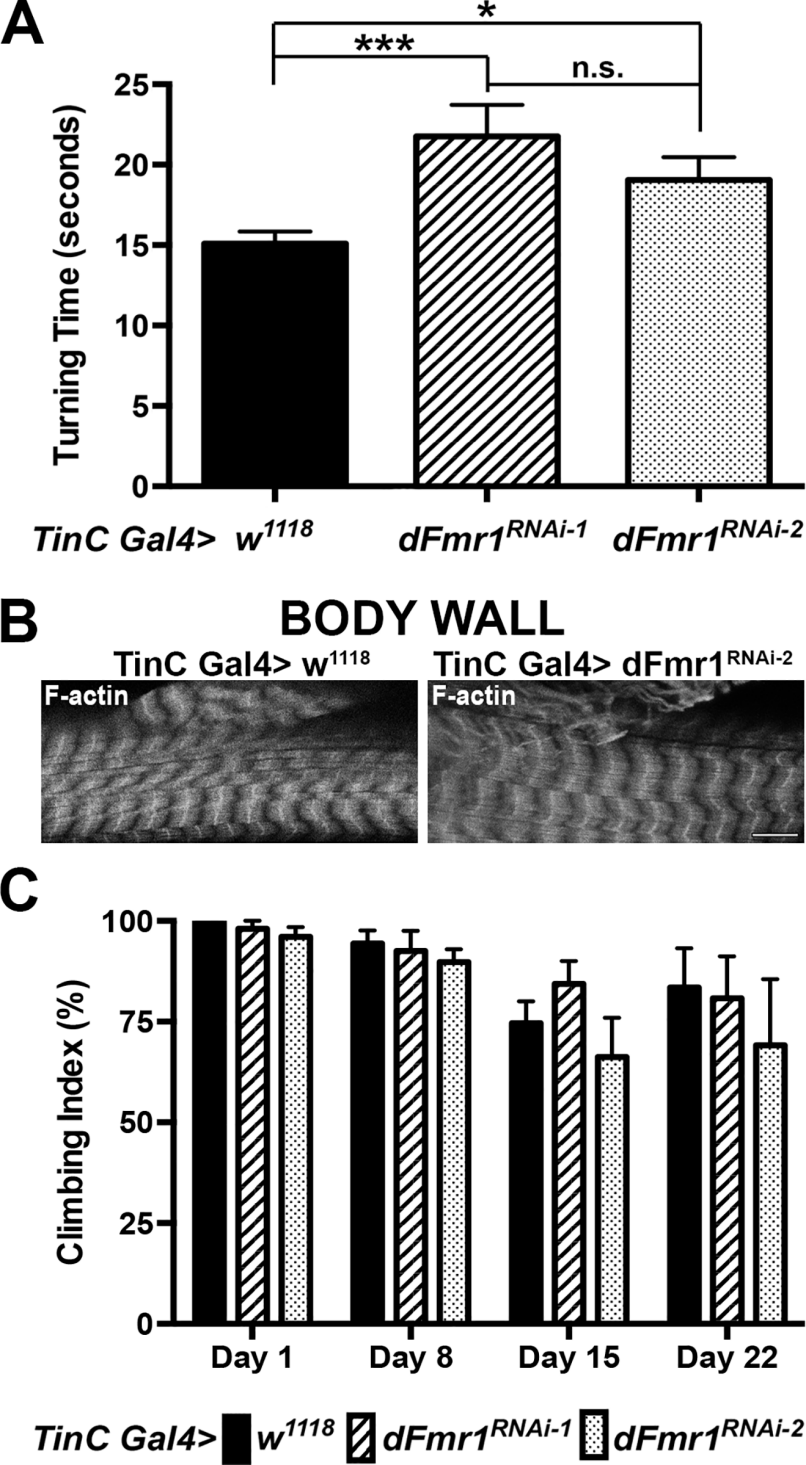
Reduced heart rate due to cardiac-specific loss of dFmr1 correlates with a developmental locomotion defect. **(A)** Heart-specific RNAi knockdown using two-independent RNAi lines, results in a significant increase in larval turning times suggesting a locomotion defect (TinC Gal4> *dFmr1* ^*RNAi-1*^, N = 30 and *dFmr1* ^*RNAi-2*^, N = 30) when compared to control (TinC Gal4> *w* ^*1118*^, N = 60). Student’s T-test was used to calculate statistical significance: p values, n.s. = no significance, * <0.05, ** <0.01, *** <0.001. **(B)** Immunofluorescence image of Texas Red phalloidin labeling of filamentous actin in the body wall muscle of the heart-specific RNAi knockdown of dFMR1 (TinC Gal4> *dFmr1* ^*RNAi-2*^). The basic structure of the body wall appears normal in the heart-specific RNAi knockdown compared to control (TinC Gal4> *w* ^*1118*^). Scale bar = 10 μm. **(C)** Adult climbing assays (4–5 replicates of 40–50 adults total performed at 25°C) show no significant difference between heart-specific RNAi knockdown (TinC Gal4> *dFmr1* ^*RNAi-1*^ or *dFmr1* ^*RNAi-2*^) compared to control (TinC Gal4> *w* ^*1118*^).

In *Drosophila*, it has been shown that both heart rate and locomotion are centrally regulated by the nervous system and temporally altered in response to stress [24–26]. Therefore, in addition to examining heart rate, we sought to identify if there are any additional physiological defects related to reduced cardiac function. We used larval turning assays, a well-established measure of locomotor behavior and function to test for additional effects on the whole organism. *dFmr1* was knocked down by RNAi in the heart using TinC Gal4 and larvae were tested for their ability to turn when dorso-ventrally challenged [19]. These experiments showed that the two-independent *dFmr1* RNAi lines both result in a significant delay in larval turning time compared to controls. Specifically, the RNAi-1 line resulted in ~1.4x delay while the RNAi-2 line results in ~1.3x delay (21.8±1.96 seconds and 19.0±1.42 seconds vs.15.1±0.75 seconds, P_value_ = 0.0031 and 0.018, **Fig 3A**). Immunofluorescence imaging of phalloidin labeled filamentous actin in the body wall muscles that harbor neuromuscular junction synapses required for coordinated movement show no obvious defects in the sarcomeric structure that might be caused by a non-autonomous effect of dFmr1 knockdown in the heart (**Fig 3B**). Given the absence of obvious structural defects and the cardiac-specific knockdown, these results suggest that the locomotion defect is likely due to reduced cardiac function. Interestingly, knocking down FMRP function in the heart with RNAi has no significant effect on adult locomotor function as measured by climbing assays (**Fig 3C**). Taken together our data suggest that the effect of FMRP knockdown in heart muscle on locomotor function, albeit indirect, is developmentally regulated.

## Discussion

Regulation of heart rate is critical for proper cardiac function. In humans, a reduction in heart rate (bradycardia) can result in poor oxygen exchange throughout the body and can lead to sudden cardiac arrest. Therefore, it is important to understand the components involved in heart rate regulation. It was previously shown that loss of Fragile X protein, FXR1 leads to perinatal lethality in mice possibly due to sudden cardiac arrest, whereas its reduction in zebrafish leads to a reduction in cardiac function [6–8]. In this present study we demonstrate that the Fragile X protein dFmr1 is essential for proper control of heart rate in *Drosophila*.

We report that loss of dFmr1, the sole Fragile X family member in *Drosophila*, results in a significant decrease in heart rate, which is reversed by restored expression of the *dFmr1* gene using a genomic rescue construct. These data indicate that dFmr1 is specifically required for proper control of heart rate and are consistent with reports in zebrafish that FXR1 loss results in reduced cardiac function [7]. Additionally, we show the decrease in heart rate is specific to cardiac muscle via the use of tissue-specific RNAi knockdown of *dFmr1* with two independent RNAi lines. These results are consistent with a cell autonomous function of dFmr1 in cardiac muscle and suggest that dFMR1 regulates cardiac-specific mRNA targets necessary for controlling heart rate.

Given that sarcomeric structures do not appear to be significantly affected by the loss of dFmr1, we speculate that its targets might encode channels or other functional determinants of contractility. In the larva, the dorsal vessel beats due to a myogenic cardiac impulse with only a pair of transverse glutamergic nerves innervating the lateral edges of the cardiac chamber [25]. Alterations in the normal electrical impulses can alter the rate of the heart’s pumping. Studies have shown that disruption of the potassium channel or the sarcoendoplasmic reticulum calcium ATPase channel in *Drosophila* results in reduced heart rate [27,28]. It is possible that dFmr1 could regulate one of these channels in the cardiomyocyte resulting in reduced heart rate.

We previously reported that FXR1 translationally regulates the desmosomal protein desmoplakin and the costameric protein talin2 in the murine heart [8]. Changes in desmosomal proteins (*i*.*e*., desmoplakin, plakophilin-2, desmoglein-2 and desmocollin) and talin have been associated with changes in heart rate [29,30]. However, *Drosophila* lacks cytoplasmic intermediate filaments (*i*.*e*., desmoplakin) and structures such as desmosomes (for review see [31]). This suggests that the heart rate change by *dFmr1* in *Drosophila* is not due to a desmosome defect but potentially due to changes in the costamere. Talin is conserved among species with *Drosophila* containing one talin gene named *rhea*. In all species, talin forms a physical link between the cytoplasmic domain of integrins and the actin cytoskeleton, thus acting as an intracellular messenger to activate integrin inside-out signaling (for review see [32]). In fact, truncation of an integrin cytoplasmic domain resulted in a loss of inside-out integrin signaling, as well as a reduction in heart rate [29]. Therefore, it is possible that in the *Drosophila* heart, dFmr1 regulates the expression of talin thus altering integrin signaling or altering the mechanical link between the cytoskeleton and the extracellular matrix, both affecting heart function. Alternatively, dFmr1 regulates mRNA targets distinct from FXR1; this possibility will be interesting to determine in the future.

We also report that cardiac-specific *dFmr1* RNAi knockdown results in a reduction of larval turning rate but has no effect on adult climbing, suggesting a developmentally regulated locomotion defect linked with a cardiac defect. The central regulation of both heart rate and locomotion in response to stress could account for the changes seen [24–26]. It is possible that the stress of a reduced heart rate leads to signals that caused a change in locomotor behavior. Additionally, although we used the cardiac specific TinC Gal4 driver to knockdown dFMR1 in the heart, and the structure of the body wall muscles that harbor neuromuscular junctions appears unaffected, a possible non-autonomous effect cannot be eliminated at this time. Indeed, it was previously shown that dFmr1 could have non-autonomous effects by affecting signaling pathways in neighboring tissue [33]. It is possible that with knockdown of dFmr1 in cardiomyocytes, the reduced cardiac function could lead to signaling that slows down larval movement, thus ensuring homeostasis.

Given the importance of Fragile X proteins in the heart, it is important to determine their role in human disease. Our results presented in this study directly implicate dFmr1 in heart rate regulation at the molecular level in cardiomyocytes, supporting the notion that Fragile X proteins play a direct role in proper cardiac function. The *Drosophila* model offers an opportunity for unbiased molecular and genetic screens that can uncover physiologically significant targets of Fragile X proteins in the heart.

## Supporting Information

S1 Fig FMRP expression Western blot. (TIF)

## References

[1] Lécuyer E, Yoshida H, Parthasarathy N, Alm C, Babak T, Cerovina T, et al (2007) Global analysis of mRNA localization reveals a prominent role in organizing cellular architecture and function. Cell 131: 174–187. 10.1016/j.cell.2007.08.003 17923096

[2] Schwanhäusser B, Busse D, Li N, Dittmar G, Schuchhardt J, Wolf J, et al (2011) Global quantification of mammalian gene expression control. Nature 473: 337–342. 10.1038/nature10098 21593866

[3] Vogel C, Marcotte EM (2012) Insights into the regulation of protein abundance from proteomic and transcriptomic analyses. Nat Rev Genet 13: 227–232. 10.1038/nrg3185 22411467 PMC3654667

[4] Kaufmann WE, Cortell R, Kau ASM, Bukelis I, Tierney E, Gray RM, et al (2004). Am J Med Genet 129A: 225–234. 10.1002/ajmg.a.30229 15326621

[5] Bassell GJ, Warren ST (2008) Fragile X syndrome: loss of local mRNA regulation alters synaptic development and function. Neuron 60: 201–214. 10.1016/j.neuron.2008.10.004 18957214 PMC3691995

[6] Mientjes EJ, Willemsen R, Kirkpatrick LL, Nieuwenhuizen IM, Hoogeveen-Westerveld M, Verweij M, et al (2004) Fxr1 knockout mice show a striated muscle phenotype: implications for Fxr1p function in vivo. Hum Mol Genet 13: 1291–1302. 10.1093/hmg/ddh150 15128702

[7] Van't Padje S, Chaudhry B, Severijnen L-A, van der Linde HC, Mientjes EJ, Oostra BA, et al (2009) Reduction in fragile X related 1 protein causes cardiomyopathy and muscular dystrophy in zebrafish. J Exp Biol 212: 2564–2570. 10.1242/jeb.032532 19648401

[8] Whitman SA, Cover C, Yu L, Nelson DL, Zarnescu DC, Gregorio CC (2011) Desmoplakin and talin2 are novel mRNA targets of fragile X-related protein-1 in cardiac muscle. Circ Res 109: 262–271. 10.1161/CIRCRESAHA.111.244244 21659647 PMC3163600

[9] Wan L, Dockendorff TC, Jongens TA, Dreyfuss G (2000) Characterization of dFMR1, a Drosophila melanogaster homolog of the fragile X mental retardation protein. Mol Cell Biol 20: 8536–8547. 11046149 10.1128/mcb.20.22.8536-8547.2000 PMC102159

[10] Ocorr K, Perrin L, Lim H-Y, Qian L, Wu X, Bodmer R (2007) Genetic control of heart function and aging in Drosophila. Trends Cardiovasc Med 17: 177–182. 10.1016/j.tcm.2007.04.001 17574126 PMC1950717

[11] Medioni C, Sénatore S, Salmand P-A, Lalevée N, Perrin L, Sémériva M (2009) The fabulous destiny of the Drosophila heart. Curr Opin Genet Dev 19: 518–525. 10.1016/j.gde.2009.07.004 19717296

[12] Wolf MJ, Rockman HA (2011) Drosophila, genetic screens, and cardiac function. Circ Res 109: 794–806. 10.1161/CIRCRESAHA.111.244897 21921272 PMC3678974

[13] Seyres D, Röder L, Perrin L (2012) Genes and networks regulating cardiac development and function in flies: genetic and functional genomic approaches. Brief Funct Genomics 11: 366–374. 10.1093/bfgp/els028 22908209

[14] Bier E, Bodmer R (2004) Drosophila, an emerging model for cardiac disease. Gene 342: 1–11. 10.1016/j.gene.2004.07.018 15527959

[15] Vigoreaux JO, Saide JD, Pardue ML (1991) Structurally different Drosophila striated muscles utilize distinct variants of Z-band-associated proteins. J Muscle Res Cell Motil 12: 340–354. 1719028 10.1007/BF01738589

[16] Wessells RJ, Bodmer R (2004) Screening assays for heart function mutants in Drosophila. BioTechniques 37: 58–60. 15283201 10.2144/04371ST01

[17] Brent JR, Werner KM, McCabe BD (2009) Drosophila Larval NMJ Dissection. JoVE. 10.3791/1107 19229190 PMC2762896

[18] Broadie KS, Bate M (1993) Development of the embryonic neuromuscular synapse of Drosophila melanogaster. J Neurosci 13: 144–166. 8093713 10.1523/JNEUROSCI.13-01-00144.1993 PMC6576301

[19] Estes PS, Boehringer A, Zwick R, Tang JE, Grigsby B, Zarnescu DC (2011) Wild-type and A315T mutant TDP-43 exert differential neurotoxicity in a Drosophila model of ALS. Hum Mol Genet 20: 2308–2321. 10.1093/hmg/ddr124 21441568 PMC3098735

[20] Brand AH, Perrimon N (1993) Targeted gene expression as a means of altering cell fates and generating dominant phenotypes. Development 118: 401–415. 8223268 10.1242/dev.118.2.401

[21] Lo PC, Frasch M (2001) A role for the COUP-TF-related gene seven-up in the diversification of cardioblast identities in the dorsal vessel of Drosophila. Mech Dev 104: 49–60. 11404079 10.1016/s0925-4773(01)00361-6

[22] Luo L, Liao YJ, Jan LY, Jan YN (1994) Distinct morphogenetic functions of similar small GTPases: Drosophila Drac1 is involved in axonal outgrowth and myoblast fusion. Genes Dev 8: 1787–1802. 7958857 10.1101/gad.8.15.1787

[23] Xiong WC, Okano H, Patel NH, Blendy JA, Montell C (1994) repo encodes a glial-specific homeo domain protein required in the Drosophila nervous system. Genes Dev 8: 981–994. 7926782 10.1101/gad.8.8.981

[24] Strauss R, Heisenberg M (1993) A higher control center of locomotor behavior in the Drosophila brain. J Neurosci 13: 1852–1861. 8478679 10.1523/JNEUROSCI.13-05-01852.1993 PMC6576564

[25] Dulcis D, Levine RB (2003) Innervation of the heart of the adult fruit fly, Drosophila melanogaster. J Comp Neurol 465: 560–578. 10.1002/cne.10869 12975816

[26] Argue KJ, Neckameyer WS (2013) Temporally dimorphic recruitment of dopamine neurons into stress response circuitry in Drosophila. Behav Neurosci 127: 725–733. 10.1037/a0033602 23895060 PMC4212825

[27] Ocorr K, Reeves NL, Wessells RJ, Fink M, Chen H-SV, Akasaka T, et al (2007) KCNQ potassium channel mutations cause cardiac arrhythmias in Drosophila that mimic the effects of aging. Proc Natl Acad Sci USA 104: 3943–3948. 10.1073/pnas.0609278104 17360457 PMC1820688

[28] Abraham DM, Wolf MJ (2013) Disruption of sarcoendoplasmic reticulum calcium ATPase function in Drosophila leads to cardiac dysfunction. PLoS ONE 8: e77785 10.1371/journal.pone.0077785 24098595 PMC3789689

[29] Valencik ML, Zhang D, Punske B, Hu P, McDonald JA, Litwin SE et al (2006) Integrin activation in the heart: a link between electrical and contractile dysfunction? Circ Res 99: 1403–1410. 10.1161/01.RES.0000252291.88540.ac 17095723

[30] Riele Te ASJM, Hauer RN (2015) Arrhythmogenic right ventricular dysplasia/cardiomyopathy: clinical challenges in a changing disease spectrum. Trends Cardiovasc Med 25: 191–198. 10.1016/j.tcm.2014.11.003 25601034

[31] Sonnenberg A, Liem RKH (2007) Plakins in development and disease. Exp Cell Res 313: 2189–2203. 10.1016/j.yexcr.2007.03.039 17499243

[32] Ciobanasu C, Faivre B, Le Clainche C (2013) Integrating actin dynamics, mechanotransduction and integrin activation: the multiple functions of actin binding proteins in focal adhesions. Eur J Cell Biol 92: 339–348. 10.1016/j.ejcb.2013.10.009 24252517

[33] Callan MA, Clements N, Ahrendt N, Zarnescu DC (2012) Fragile X Protein is required for inhibition of insulin signaling and regulates glial-dependent neuroblast reactivation in the developing brain. Brain Res 1462: 151–161. 10.1016/j.brainres.2012.03.042 22513101

