# Supplementary figures and images for: Regulation of Heart Rate in Drosophila via Fragile X Mental Retardation Protein
Source: PLoS One. 2015 Nov 16;10(11):e0142836. doi: 10.1371/journal.pone.0142836 (PMC4646288; doi:10.1371/journal.pone.0142836)

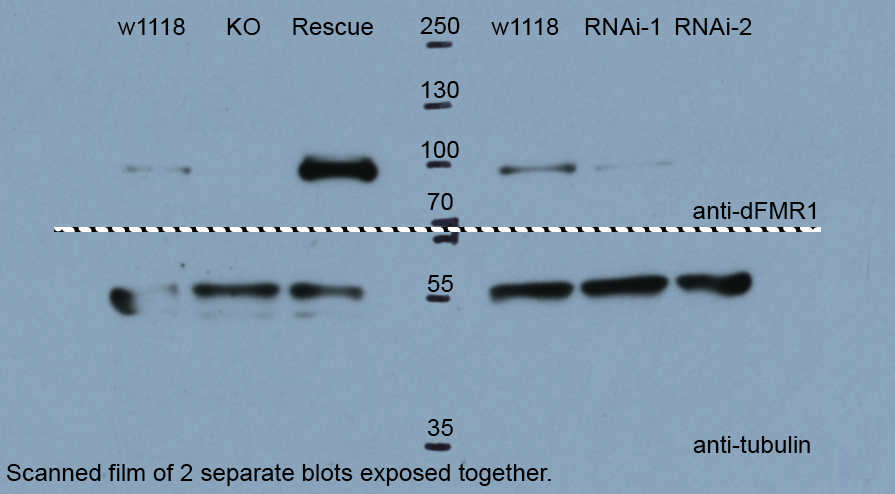

Supplement: S1 Fig — (TIF) [file pone.0142836.s001.tif]
